# Circulating Endotrophin Predicts Myocardial Fibrosis Burden and Is Sensitive to Antifibrotic Therapy

**DOI:** 10.1016/j.jacadv.2026.102738

**Published:** 2026-04-17

**Authors:** Nicholas Black, Gavin Lewis, Fardad Soltani, Susanna Dodd, Simon G. Williams, Erik B. Schelbert, Clara Laursen, Elisavet Angeli, Alexander L. Reese-Petersen, Morten Karsdal, Federica Genovese, Christopher A. Miller

**Affiliations:** a Division of Cardiovascular Sciences, School of Medical Sciences, Faculty of Biology, Medicine and Health, Manchester Academic Health Science Centre, University of Manchester, Manchester, United Kingdom; b Manchester University NHS Foundation Trust, Manchester, United Kingdom; c Liverpool University Hospitals NHS Foundation Trust, Royal Liverpool University Hospital, Liverpool, United Kingdom; d Department of Health Data Science, Institute of Population Health, Faculty of Health and Life Sciences, University of Liverpool, Liverpool, United Kingdom; e Allina Health Minneapolis Heart Institute, Abbott Northwestern Hospital, Minneapolis, Minnesota, USA; f Department of Biomedical Sciences, University of Copenhagen, Copenhagen, Denmark; g Nordic Bioscience A/S, Herlev, Denmark

**Keywords:** cardiovascular magnetic resonance, endotrophin, extracellular volume, HFpEF, myocardial fibrosis, pirfenidone

## Abstract

**Background:**

Novel collagen-derived circulating peptides, such as endotrophin, have been proposed as biomarkers of myocardial fibrosis.

**Objectives:**

We aimed to determine the effect of pirfenidone, an antifibrotic agent, on circulating levels of these peptides, and their association with cardiovascular magnetic resonance extracellular volume (ECV).

**Methods:**

In the PIROUETTE (Pirfenidone in Patients with Heart Failure and Preserved Left Ventricular Ejection Fraction) trial, novel collagen-derived circulating peptides (PRO-C3, C3M, CTX-III, endotrophin, PRO-C6, and C6M) were measured at baseline and at prespecified time points in patients with ECV ≥27% randomized (n = 94) to pirfenidone or placebo. Baseline peptide levels were also measured in patients with ECV <27% who were not randomized (n = 13).

**Results:**

Treatment with pirfenidone was associated with a significant reduction in log endotrophin (*P* = 0.034), with a treatment effect seen from 13 weeks. After multivariable adjustment there were significant albeit modest associations between change in myocardial ECV and change in log endotrophin (R^2^: 0.14; *P* = 0.031), and baseline ECV and baseline log endotrophin (R^2^: 0.30; *P* = 0.022). Pirfenidone had no effect on the levels of other collagen-derived circulating peptides, and there were no associations between their levels and change in myocardial ECV or baseline ECV.

**Conclusions:**

In patients with heart failure with preserved ejection fraction, treatment with pirfenidone was associated with a sustained reduction in circulating levels of endotrophin from 13 weeks. Circulating endotrophin was also independently associated with both baseline myocardial ECV and change in myocardial ECV. Endotrophin shows high potential as a circulating biomarker reflective of myocardial fibrosis burden and sensitive to change in myocardial fibrosis over time.

Myocardial fibrosis is a key pathophysiological mechanism that is increasingly recognized as an important therapeutic target in many cardiovascular diseases.^1^ In patients with heart failure (HF) with preserved ejection fraction (HFpEF), myocardial fibrosis, measured using cardiovascular magnetic resonance (CMR) extracellular volume (ECV), is independently predictive of load-independent intrinsic left ventricular stiffness and adverse outcome, including hospitalization for HF and death.^2^^,^^3^ There is growing interest in noninvasive measurement of myocardial fibrosis, to guide prognosis and identify patients who may benefit from targeted antifibrotic therapy. CMR ECV represents the best validated noninvasive measure of myocardial fibrosis,^4^^,^^5^ but CMR remains relatively uncommon and costly.

Circulating biomarkers of myocardial fibrosis would represent cheaper and more accessible alternatives.^6^ The ideal circulating biomarker would be specific to the myocardium, strongly correlate with histological or CMR-derived myocardial fibrosis, and be dynamically sensitive to change in myocardial fibrosis burden, including in response to antifibrotic therapy.^6^

Circulating collagen type I and III-derived peptides have been extensively investigated as potential biomarkers of myocardial fibrosis, but results have been inconsistent. Recent advances in assay technology have enabled the detection of a broader range of collagen-derived peptides, offering more comprehensive evaluation of collagen metabolism. Specifically, assays targeting type III collagen turnover include markers of synthesis (PRO-C3), degradation (C3M), and cross-linked fibrinolysis (CTX-III). Similarly, assays targeting type VI collagen turnover reflect synthesis (endotrophin and PRO-C6) and degradation (C6M). These biomarkers have been shown to correlate with fibrosis in a wide variety of tissues, including liver,^7^, ^8^, ^9^, ^10^ lungs,^11^^,^^12^ and kidney.^13^^,^^14^ However, their association with myocardial fibrosis, as measured by myocardial ECV, and response to antifibrotic therapy, has not been investigated.

Pirfenidone, an oral antifibrotic drug licensed for idiopathic pulmonary fibrosis, mediates antifibrotic effects by inhibiting transforming growth factor-beta and fibroblast proliferation and differentiation.^15^ In the PIROUETTE (Pirfenidone in Patients with Heart Failure and Preserved Left Ventricular Ejection Fraction) trial of patients with HFpEF and evidence of myocardial fibrosis (ECV ≥27%), pirfenidone reduced myocardial fibrosis, as measured using CMR ECV.^16^

In this study, we aimed to investigate the relationship between novel circulating collagen-derived peptides (PRO-C3, C3M, CTX-III, endotrophin, PRO-C6, and C6M) and CMR ECV, and the impact of pirfenidone.

## Methods

### Trial design

Between March 7, 2017, and December 19, 2018, the PIROUETTE trial (NCT02932566) randomized 94 patients with HFpEF and myocardial fibrosis to pirfenidone or placebo for 52 weeks. The trial design and results have previously been published.^16^^,^^17^ Eligibility requirements included age ≥40 years, symptoms and signs of HF, left ventricular ejection fraction ≥45%, and elevated plasma concentrations of natriuretic peptides (brain-type natriuretic peptide ≥100 pg/mL or N-terminal pro-B-type natriuretic peptide [NT-proBNP] ≥300 pg/mL; or brain-type natriuretic peptide ≥300 pg/mL or NT-proBNP ≥900 pg/mL if in atrial fibrillation). Eligible patients underwent CMR and those with evidence of myocardial fibrosis, defined as an ECV of ≥27%, were randomized in a 1:1 ratio to treatment with either pirfenidone 2403 mg daily or matching placebo for 52 weeks using block randomization, stratified by sex. Patients with ECV <27% were entered into a registry (n = 13). Key exclusion criteria included alternative causes of patients' symptoms such as pulmonary disease, anemia, or obesity; pericardial constriction, hypertrophic cardiomyopathy (HCM), or infiltrative cardiomyopathy; and contraindications to magnetic resonance imaging. The primary outcome was change in myocardial fibrosis, measured using CMR ECV, from baseline to 52 weeks.

The trial was sponsored by Manchester University NHS Foundation Trust and Liverpool Clinical Trials Centre, a United Kingdom Clinical Research Collaboration Registered Clinical Trials Unit, was the clinical trials unit. The investigational medicinal product was gifted by Roche Products Limited. Roche Products Limited had no role in study design or data analysis. The study protocol was approved by a research ethics committee and trial conduct was overseen by a trial steering committee. Patients were identified at 6 hospitals in the United Kingdom. Study visits took place at Manchester University NHS Foundation Trust. All patients provided written informed consent.

### Circulating collagen-derived peptide analysis

Patients provided written informed consent for blood samples to be stored in a central biorepository at baseline and at 13-, 26-, and 52-weeks post randomization. Plasma was stored at −80-degrees and transported on dry ice to Nordic Bioscience, Denmark. The sample assessment was performed using enzyme-linked immunosorbent assays following the manufacturer’s protocols (Nordic Bioscience A/S). More specifically, the samples were analyzed for 6 novel collagen peptides with the following assays: nordicPRO-C3 targeting the N-terminal procollagen propeptide released during type III collagen synthesis;^18^ nordicC3M targeting the type III collagen breakdown product by matrix metalloproteinase-9;^8^ nordicCTX-III targeting the cross-linked type III collagen fibrinolysis product^9^; nordicPRO-C6 and nordicEndotrophin targeting the C-terminal procollagen propeptide released during type VI collagen synthesis, corresponding to the C-terminal end of the signaling molecule endotrophin, and the full-length endotrophin molecule,^19^^,^^20^ respectively; and nordicC6M targeting the type VI collagen breakdown product by matrix metalloproteinase-2 and -9. The assays were carried out according to the manufacturers’ instructions as previously described.

### Statistical analysis

The original trial design included prospective sample storage at baseline 13, 26, and 52 weeks for unspecified future analyses. Fibrosis biomarkers were not included in the original Statistical Analysis Plan and thus the analyses included in this study are considered post hoc. The trial was not powered for secondary outcomes; thus, the findings of this study are exploratory. No adjustments were made for multiple comparisons in view of the exploratory nature of these analyses.

Continuous data are presented as mean (SD) or median [IQR], as appropriate. Categorical data are presented as counts and percentages. The distributions of collagen biomarkers were non-normal (Supplemental Figure 1) (Shapiro-Wilk tests for normality were *P* < 0.001). Data were logarithmically transformed to normalize the data (Supplemental Figure 2).

Treatment-related analyses were conducted on an intention-to-treat basis. Biomarker levels at week 52 were compared between treatment groups using analysis of covariance (ANCOVA), adjusting for baseline levels, stratification factor (sex), and treatment group. Power analysis (G∗Power) demonstrated that 94 randomized patients achieved a statistical power of 67% to predict a medium effect size (Cohen f 0.25) with a type 1 error rate of 0.05.^21^ Missing data for the ANCOVA analysis were handled using complete case analysis. The impact of pirfenidone treatment on biomarker levels over time was also determined using a repeated-measure linear mixed model with an unstructured covariance structure. Time (months), baseline levels, sex, treatment group and interaction between time and treatment group were included as fixed effects. Biomarker levels at different time points were compared using ANCOVA as described previously.

Univariable and multivariable regression models were used to assess the relationships between change in myocardial ECV from baseline to week 52, and change in biomarker levels. Variables for which *P* values were <0.3 in the univariable analyses proceeded to combined forward and reverse stepwise Akaike Information Criterion (AIC) selection. The chosen variables were then included in multivariable regression models, alongside collagen biomarkers. The same methods were also used to assess the relationship between baseline myocardial ECV, and baseline biomarkers. Baseline biomarker levels between randomized (ECV ≥27%) and registry (ECV <27%) were compared using a 2-sample t test.

## Results

### Patient characteristics

Ninety-four patients with evidence of myocardial fibrosis (ECV ≥27%) were randomized to receive pirfenidone or placebo. A further 13 patients without myocardial fibrosis (ECV <27%) were enrolled into a registry. Baseline characteristics are summarized in Table 1. At the end of the trial, 12 of the 94 patients who were randomized had withdrawn from the study and 2 had died. No patients were lost to follow-up. At baseline, plasma samples were available for 93 (of 94) randomized patients and 13 (of 13) registry patients. During follow-up, plasma samples were available for 83, 61, and 80 randomized patients at 13, 26, and 52 weeks, respectively.

**Table 1:** Table of Baseline Characteristics

	Randomized (ECV ≥27%)		Registry (ECV <27%, n = 13)
	Placebo (n = 47)	Pirfenidone (n = 47)	
Age (y)	79.5 (6.2)	76.2 (8.1)	73.9 (8.3%)
Female (%)	21 (44.7%)	22 (46.8%)	9 (69.2%)
White ethnicity (%)	43 (91.5%)	45 (95.7%)	13 (100%)
BMI (kg/m^2^)	30.4 (6.1)	31.4 (5.2)	33.8 (4.5)
NYHA functional class (%)			
I	5 (10.6%)	0 (0.0%)	1 (7.7%)
II	19 (40.4%)	26 (55.3%)	11 (84.6%)
III	23 (48.9%)	21 (44.7%)	1 (7.69%)
IV	0 (0.0%)	0 (0.0%)	0 (0.0%)
Comorbidities			
Hypertension (%)	40 (85.1%)	39 (83.0%)	10 (76.9%)
Diabetes (%)	12 (25.5%)	16 (34.0%)	2 (15.4%)
Atrial fibrillation (%)	27 (57.4%)	27 (57.4%)	4 (30.8%)
Stroke (%)	5 (10.6%)	5 (10.6%)	1 (7.7%)
Hyperlipidemia (%)	12 (25.5%)	10 (21.3%)	1 (7.7%)
Ischemic heart disease (%)	19 (40.4%)	17 (36.2%)	4 (30.8%)
Prior HF hospitalization (%)	7 (14.9%)	8 (17.0%)	0 (0.0%)
COPD (%)	7 (14.9%)	5 (10.6%)	0 (0.0%)
Current smoker (%)	0 (0.0%)	1 (2.13%)	0 (0.0%)
Exsmoker (%)	17 (36.2%)	15 (31.9%)	8 (61.5%)
Laboratory measurements			
Hemoglobin (g/dL)	12.8 (1.5)	13.1 (1.5)	13.5 (1.1)
White cell count (10^9^/L)	7.9 (2.3)	7.9 (2.2)	7.5 (1.8)
Sodium (mmol/L)	138.4 (3.0)	138.6 (3.7)	140.0 (3.7)
Creatinine (umol/L)	110.0 (32.8)	98.5 (23.4)	97.9 (25.7)
eGFR (ml/min/1.73 m^2^)	54.2 (17.2)	59.6 (15.9)	57.5 (16.4)
HsTropT (pg/mL)	25.7 [15.3-38.0]	17.0 [11.0-24.7]	13.3 [10.0-17.0]
NT-proBNP (pg/mL)	1372.0 [626.0-2817.0]	975.0 [445.0-2064.0]	423 [324.0-803.0]
log NT-proBNP	7.2 [6.4-7.9]	6.9 [6.1-7.6]	6.0 [5.8-6.7]
GDF-15 (pg/mL)	3046.0 [1970.0-5422.0]	2388.0 [1749.0-3116.0]	1724.0 [1563.0-1954.0]
log GDF-15	8.0 [7.6-8.6]	7.8 [7.5-8.0]	7.5 [7.4-7.6]
PRO-C3 (ng/mL)	96.6 [68.5-137.6]	81.9 [60.6-108.0]	58.2 [53.5-66.4]
log PRO-C3	4.6 [4.2-4.9]	4.4 [4.1-4.7]	4.1 [4.0-4.2]
C3M (ng/mL)	516.0 [467.0-588.5]	521.0 [467.0-602.3]	575.0 [427.0-627.0]
log C3M	6.2 [6.1-6.4]	6.3 [6.1-6.4]	6.4 [6.1-6.4]
CTX-III (ng/mL)	12.2 [5.7-28.1]	6.1 [5.5-24.2]	10.5 [5.5-18.5]
log CTX-III	2.5 [1.7-3.3]	1.8 [1.7-3.2]	2.4 [1.7-2.9]
Endotrophin (ng/mL)	158.0 [119.2-193.5]	126.9 [103.7-155.5]	132.9 [103.3 -187.6]
log endotrophin	5.1 [4.8-5.3]	4.8 [4.6-5.0]	4.9 [4.6-5.2]
PRO-C6 (ng/mL)	17.5 [13.6-22.1]	15.1 [12.3-19.5]	12.6 [11.7-18.1]
log PRO-C6	2.9 [2.6-3.1]	2.7 [2.5-3.0]	2.5 [2.5-2.9]
C6M (ng/mL)	896.0 [810.0-1076.0]	859.0 [762.8-951.5]	845.0 [825.0-910.0]
log C6M	6.8 [6.7-7.0]	6.8 [6.6-6.9]	6.7 [6.7-6.8]
Cardiac MRI measurements			
LVMassi (g/m^2^)	66.8 (17.4)	62.9 (13.1)	66.8 (13.6)
LVEDVi (mL/m^2^)	62.2 (18.4)	62.9 (18.8)	67.4 (10.8)
LVESVi (mL/m^2^)	23.9 (10.9)	22.5 (11.1)	21.2 (7.3)
LVEF (%)	62.8 (9.1)	65.2 (8.1)	69.0 (8.2)
RVEDVi (mL/m^2^)	68.0 (16.2)	69.6 (16.7)	72.3 (12.5)
RVESVi (mL/m^2^)	34.9 (9.8)	32.9 (9.7)	30.5 (8.7)
RVEF (%)	50.6 (9.9)	53.1 (8.9)	57.1 (9.4)
LAVi (mL/m^2^)	71.2 (19.4)	69.6 (18.0)	62.1 (13.7)
RAVi (mL/m^2^)	70.5 (27.0)	71.4 (27.2)	49.3 (17.2)
Aortic distensibility (10^-3^/mm Hg)	1.6 (0.8)	1.6 (1.0)	1.1 (0.7)
Infarct LGE (%)	12 (25.5%)	8 (17%)	0 (0.0%)
Nonischemic LGE (%)	18 (38.3%)	12 (25.5%)	1 (7.7%)
Myocardial ECV (%)	30.7 (2.9)	29.5 (2.5)	24.7 (0.6)

Values are mean (SD), or median [IQR].

BMI = body mass index; COPD = chronic obstructive pulmonary disease; ECV = extracellular volume; eGFR = estimated glomerular filtration rate; GDF-15 = growth differentiation factor 15; HF = heart failure; LAVi = indexed left atrial volume; HsTropT = high sensitivity troponin T; LGE = late gadolinium enhancement; LVEDVi = indexed left ventricle end-diastolic volume; LVESVi = indexed left ventricle end-systolic volume; LVEF = left ventricle ejection fraction; LVMassi = indexed left ventricle mass; NT-proBNP = N-terminal pro B-type natriuretic peptide; MRI = magnetic resonance imaging; RAVi = indexed right atrial volume; RVEDVi = indexed right ventricle end diastolic volume; RVESVi = indexed right ventricle end systolic volume; RVEF = right ventricle ejection fraction.

### Effect of pirfenidone on circulating collagen-derived peptides

**Figure 1:**
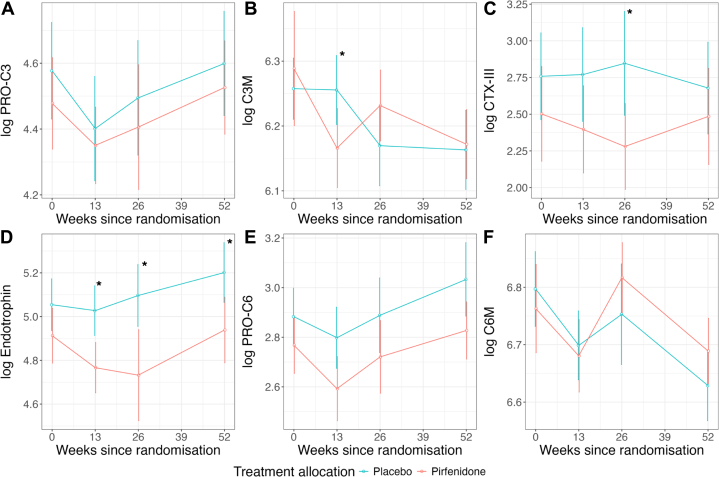
**Collagen Biomarker Levels During Follow-Up** Levels of (A) log PRO-C3, (B) log C3M, (C) log CTX-III, (D) log endotrophin, (E) log PRO-C6, and (F) log C6M, from baseline to 52 weeks in patients randomized to pirfenidone and placebo. Values are mean ± 95% CIs. ∗*P* < 0.05.

Levels of circulating biomarkers in patients randomized to pirfenidone or placebo are shown in Figure 1 and Table 2. Treatment with pirfenidone significantly reduced levels of endotrophin at week 52 (log endotrophin mean and 95% CI: 5.20 (4.34-6.06) vs 4.94 (4.00-5.88), for placebo vs pirfenidone; ANCOVA *P* = 0.034) (Supplemental Table 1), with a treatment effect seen by week 13 (*P* = 0.002) and which persisted thereafter (week 26; *P* = 0.007) (Figure 1E). Pirfenidone treatment had no effect on levels of the other biomarkers at week 52 (Supplemental Table 1). The results were unchanged after additional adjustment for body mass index and estimated glomerular filtration rate (Supplemental Table 2). Repeated measures linear mixed modeling demonstrated that pirfenidone treatment was associated with a significant reduction in log endotrophin (*P* = 0.014), but the interaction between time and pirfenidone treatment group was nonsignificant (*P* = 0.858) (Supplemental Table 3). No effect was seen for the other biomarkers (Supplemental Table 3).

**Table 2:** Circulating Levels of Novel Collagen Biomarkers in Patients Randomized to Pirfenidone or Placebo

	Week 0			Week 13			Week 26			Week 52		
	Placebo	Pirfenidone	*P* Value	Placebo	Pirfenidone	*P* Value	Placebo	Pirfenidone	*P* Value	Placebo	Pirfenidone	*P* Value
log PRO-C3	4.58 (0.51)	4.48 (0.48)	0.344	4.40 (0.54)	4.35 (0.37)	0.891	4.49 (0.52)	4.41 (0.49)	0.611	4.60 (0.52)	4.53 (0.45)	0.970
log C3M	6.26 (0.17)	6.29 (0.30)	0.546	6.26 (0.18)	6.17 (0.19)	0.028	6.17 (0.18)	6.23 (0.15)	0.203	6.16 (0.20)	6.17 (0.17)	0.711
log CTX-III	2.76 (1.03)	2.50 (1.12)	0.256	2.77 (1.08)	2.40 (0.95)	0.341	2.85 (1.02)	2.28 (0.78)	0.019	2.68 (1.02)	2.48 (1.05)	0.073
log endotrophin	5.05 (0.41)	4.91 (0.43)	0.114	5.03 (0.38)	4.77 (0.37)	0.002	5.10 (0.42)	4.73 (0.55)	0.007	5.20 (0.44)	4.94 (0.48)	0.034
log PRO-C6	2.88 (0.40)	2.77 (0.39)	0.170	2.80 (0.42)	2.59 (0.41)	0.238	2.89 (0.45)	2.72 (0.39)	0.358	3.03 (0.48)	2.83 (0.37)	0.213
log C6M	6.80 (0.23)	6.76 (0.27)	0.515	6.70 (0.20)	6.68 (0.20)	0.614	6.75 (0.25)	6.82 (0.16)	0.279	6.63 (0.20)	6.69 (0.18)	0.176

Values are mean (SD).

*P* values for pirfenidone treatment group in ANCOVA adjusted for baseline biomarker level (with exception at week 0), sex, and pirfenidone treatment group.

### Association between change in myocardial ECV and change in circulating collagen-derived peptides

**Figure 2:**
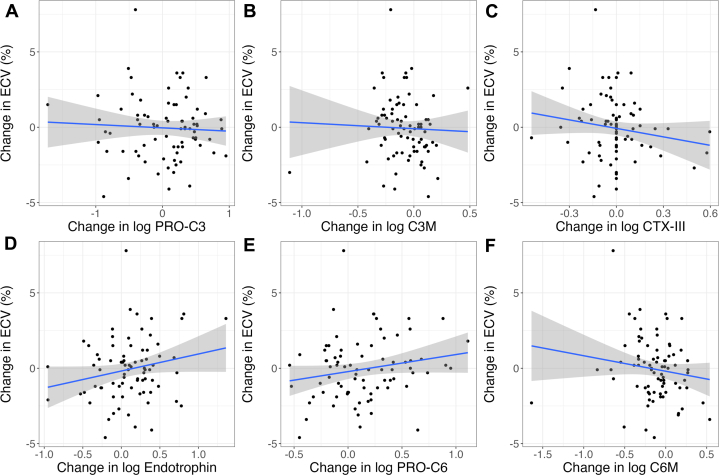
**Univariable Associations Between Change in Extracellular Volume and Collagen Biomarkers** Univariable associations between change in ECV and change in (A) log PRO-C3, (B) log C3M, (C) log CTX-III, (D) log endotrophin, (E) log PRO-C6, and (F) log C6M from baseline to week 52. Regression line (blue) and 95% CI (gray). ECV = extracellular volume.

No significant univariable associations were noted between change in myocardial ECV from baseline to week 52 and change in circulating biomarkers (Figure 2, Supplemental Table 4). After multivariable adjustment, there was a significant association between change in myocardial ECV and change in log endotrophin (R^2^ 0.14, *P* = 0.031) (Table 3 and Supplemental Table 5), but not for the other biomarkers (Table 3, Supplemental Tables 6-10). Endotrophin provided modest incremental benefit in explaining the variance in change in ECV (base model R^2^ 0.10 vs base model with endotrophin R^2^ 0.14, F-test *P* = 0.031) (Supplemental Table 5). A 1 SD increase in log endotrophin from baseline to week 52 was associated with an increase in ECV of 0.5% (Table 3).

**Table 3:** Multivariable Adjusted Associations Between Change in Myocardial ECV and Change in Circulating Biomarkers From Baseline to Week 52

Variable	Multivariable Associations			
	Regression Coefficient (SE)	T Statistic	*P* Value	Adjusted R^2^
Change in log PRO-C3^a^	−0.21 (0.23)	−0.92	0.362	0.10
Change in log C3M^a^	−0.13 (0.24)	−0.56	0.577	0.09
Change in log CTX-III^a^	−0.34 (0.22)	−1.55	0.126	0.11
Change in log endotrophin^a^	0.50 (0.23)	2.21	0.031	0.14
Change in log PRO-C6^a^	0.38 (0.22)	1.72	0.090	0.11
Change in log C6M^a^	−0.21 (0.23)	−0.90	0.373	0.10

a Regression coefficients standardized to 1 SD change in continuous variables.

### Association between baseline myocardial ECV and circulating collagen-derived peptides

**Figure 3:**
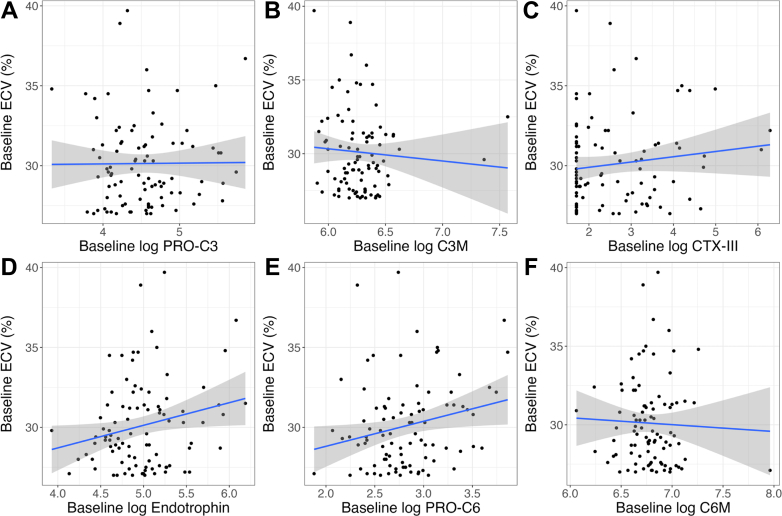
**Univariable Associations Between Baseline Extracellular Volume and Collagen Biomarkers** Univariable associations between baseline ECV and baseline (A) log PRO-C3, (B) log C3M, (C) log CTX-III, (D) log endotrophin, (E) log PRO-C6, and (F) log C6M. Regression line (blue) and 95% CI (gray). Abbreviation as in Figure 2.

Significant univariable associations were noted between baseline myocardial ECV and baseline levels of log endotrophin and log PRO-C6 (Figure 3, Supplemental Table 11). After multivariable adjustment, there was a significant association between baseline myocardial ECV and baseline log Endotrophin (R^2^ 0.30, *P* = 0.022, Table 4 and Supplemental Table 12), but not for the other biomarkers (Table 4, Supplemental Tables 13 to 17). Endotrophin provided modest incremental benefit in explaining the variance in ECV (base model R^2^ 0.26 vs base model with endotrophin R^2^ 0.30, F-test *P* = 0.022) (Supplemental Table 12). A 1 SD increase in baseline log endotrophin was associated with an increase in baseline ECV percentage of 0.6% (Table 4).

**Table 4:** Multivariable Adjusted Associations Between Baseline Myocardial ECV and Baseline Circulating Biomarkers

	Multivariable Associations			
	Regression Coefficient (SE)	T Statistic	*P* Value	Adjusted R^2^
log PRO-C3^a^	−0.06 (0.26)	−0.24	0.813	0.27
log C3M^a^	0.23 (0.26)	0.87	0.385	0.28
log CTX-III^a^	0.08 (0.27)	0.29	0.770	0.28
log endotrophin^a^	0.60 (0.26)	2.33	0.022	0.30
log PRO-C6^a^	0.30 (0.29)	1.03	0.306	0.29
log C6M^a^	0.16 (0.27)	0.60	0.554	0.28

a Regression coefficients standardized to 1 SD change in continuous variables.

Baseline levels of log PRO-C3 were significantly higher in patients with ECV ≥27% compared to ECV <27% (*P* = 0.004) (Supplemental Table 18). There were no differences in baseline levels of other biomarkers in patients with ECV ≥27% compared to ECV <27% (Supplemental Table 18).

## Discussion

In this work, we demonstrate that in patients with HFpEF and evidence of myocardial fibrosis, treatment with pirfenidone was associated with a reduction in endotrophin, with an effect seen as early as 13 weeks after commencing treatment (Central Illustration). Furthermore, endotrophin levels were independently associated with both baseline myocardial ECV and change in myocardial ECV (Central Illustration). These findings suggest that endotrophin may be a promising circulating biomarker that not only reflects the burden of myocardial fibrosis but also responds dynamically to changes in fibrosis over time.

**Central Illustration:**
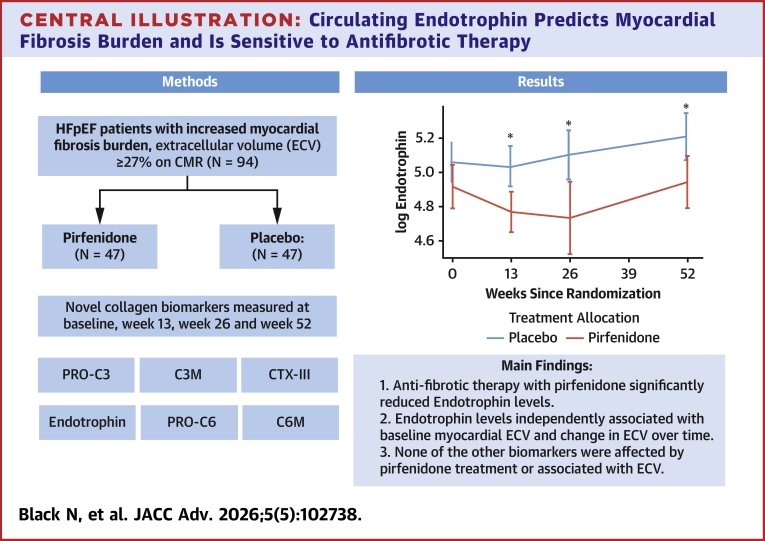
**Circulating Endotrophin Predicts Myocardial Fibrosis Burden and Is Sensitive to Antifibrotic Therapy** ECV = extracellular volume; HFpEF = heart failure with preserved ejection fraction.

Myocardial fibrosis is characterized by the excessive deposition of extracellular matrix proteins, particularly collagen, by cardiac fibroblasts, with disruption of myocardial architecture.^22^ Myocardial collagen content is largely composed of type I (∼85%) and type III (∼10%) collagens.^23^ These fibrillar collagens are located within connective tissue bundles within the myocardium (perimysium and endomysium), providing tensile strength and elasticity, respectively.^24^ In recent years, there has been a growing interest in the role of nonfibrillar collagens in myocardial fibrosis.^25^ Type VI collagen, accounting for ∼5% of myocardial collagen content, is a beaded nonfibrillar collagen that plays a critical role in anchoring components of the cellular basement membrane (such as type IV collagen and fibronectin) to the surrounding extracellular matrix.^23^ Accumulation of type VI collagen has been implicated in myocardial fibrosis, acting as a potent inducer of cardiac fibroblast differentiation into activated myofibroblasts.^26^ Type VI collagen and myofibroblast levels are significantly elevated from infarct zones in rat models.^26^ Furthermore, type VI collagen knockout mice (Col6a1−/−) exhibit smaller infarct size and improved ejection fraction compared to wild-type controls following myocardial infarction.^27^ In patients with HCM, increased type VI collagen abundance on endomyocardial biopsy correlates with invasive measures of systolic and diastolic impairment.^28^

There is significant interest in developing circulating biomarkers of myocardial fibrosis to guide prognosis and identify patients who may benefit from antifibrotic therapy. To date, no circulating biomarkers have been identified that are specific to the myocardium, strongly correlate with histological or CMR-derived myocardial fibrosis, and dynamically respond to changes in myocardial fibrosis burden.

Established circulating markers of type I and type III collagen synthesis, such as procollagen type-I C-terminal propeptide (PICP) and procollagen type-III N-terminal pro-peptide, show positive, but inconsistent, correlations with the histologically-assessed collagen volume fraction (R^2^ 0.22-0.77).^29^, ^30^, ^31^ Markers of type I collagen degradation such as collagen type-1 C-terminal telopeptide (CITP), have shown both positive^29^ and negative relationships with collagen volume fraction.^32^ In patients with HCM, no significant relationship was noted between PICP, CITP, or PICP:CITP ratio and late gadolinium enhancement, an imaging biomarker of focal myocardial fibrosis.^33^ Findings such as these have led to a call for a reappraisal of these established biomarkers, and a search for more robust circulating biomarkers of myocardial fibrosis.^6^

Endotrophin is the extracellular cleavage product derived from the C5 terminal domain of the type VI collagen alpha-3 chain.^34^ In previous studies, endotrophin levels have typically been assessed using the PRO-C6 assay, which uses a monoclonal antibody targeting the last 10 amino acids of the alpha-3 chain. However, this assay is not specific for the full-length 77 amino acid long endotrophin molecule, as it also measures other type VI collagen propeptide fragments of variable length.^35^ In the present study, endotrophin was quantified using both the traditional PRO-C6 assay and a newly developed sandwich assay employing two antibodies that bind the full-length endotrophin molecule at both ends.

Endotrophin, quantified by PRO-C6, has shown a strong prognostic biomarker profile in multiple HFpEF cohorts, where it was associated with higher risk of mortality and hospitalization for HF even after adjustment for NT-proBNP and Meta-Analysis Global Group in Chronic (MAGGIC) Heart Failure risk score.^36^ In addition, endotrophin has been shown to induce fibrosis, inflammation and insulin resistance,^37^ and directly stimulate collagen production by cardiac fibroblasts.^38^

The primary finding of this study was that treatment with pirfenidone led to a significant and sustained reduction in circulating endotrophin levels from week 13 through week 52 (Figure 1). In addition, endotrophin levels were independently associated with both baseline myocardial ECV and its change over time, although the coefficients of determination (R^2^) for these associations were modest (Tables 3 and 4). No significant associations were observed for the other novel collagen biomarkers.

These findings have several important implications. Type VI collagen is widely expressed in various tissues (eg skin, skeletal muscle, kidney, and adipose tissue), hence circulating Endotrophin levels are not specific to the myocardium.^34^ However, the independent association between circulating endotrophin levels and baseline myocardial ECV suggests that circulating levels are at least partly reflective of myocardial fibrosis burden. Although baseline endotrophin levels were similar between randomized patients with ECV ≥27% and registry patients with ECV <27%, these groups differed with respect to baseline characteristics other than myocardial ECV (Table 1). In addition, the observed correlation between changes in myocardial ECV and changes in circulating endotrophin following pirfenidone treatment suggests that changes in circulating endotrophin may partially reflect a direct effect of pirfenidone on type VI collagen metabolism within the heart, beyond its systemic antifibrotic effects. Importantly, a substantial proportion of the variance in ECV remained unexplained, highlighting that further biomarkers are needed to accurately quantify myocardial fibrosis burden.

### Study Limitations

This study has several limitations. All analyses were post hoc and performed on a small sample size; hence, the study was underpowered and should be considered as hypothesis-generating. No adjustments were made for multiple comparisons in view of the exploratory nature of these analyses. Larger, adequately powered, studies are required. ECV is an imperfect surrogate of fibrosis and may be affected by things other than collagen deposition, such as edema. This study focused on measuring only 6 novel collagen-derived peptides; other promising circulating biomarkers of fibrosis, such as galectin-3, were not investigated.^39^^,^^40^

## Conclusions

In patients with HFpEF and evidence of myocardial fibrosis, treatment with pirfenidone was associated with a sustained reduction in circulating levels of endotrophin, with the effect seen from 13 weeks. Furthermore, circulating endotrophin was independently associated with both baseline myocardial ECV and change in myocardial ECV. Endotrophin may be a promising circulating biomarker that not only reflects the burden of myocardial fibrosis but also responds dynamically to changes in fibrosis over time.

Perspectives **COMPETENCY IN MEDICAL KNOWLEDGE:** Endotrophin, a collagen type VI-derived peptide, is a promising biomarker of myocardial fibrosis. This analysis demonstrated that in patients with HFpEF and myocardial fibrosis, antifibrotic therapy with pirfenidone reduced circulating levels of endotrophin. Circulating endotrophin was independently associated with both baseline myocardial fibrosis burden and change in myocardial fibrosis.

**TRANSLATIONAL OUTLOOK:** This study’s findings require validation in larger adequately powered studies. Circulating biomarkers of myocardial fibrosis would represent cheaper and more accessible alternatives to costly imaging modalities.

## Funding support and author disclosures

The views expressed in this publication are those of the authors and not necessarily those of the NIHR, NHS or the UK Department of Health and Social Care. Dr Black, British Heart Foundation Clinical Research Training Fellow, FS/CRTF24/24601, is funded by the 10.13039/501100000274British Heart Foundation (BHF). Prof Miller, Advanced Fellowship, NIHR301338, is funded by the 10.13039/501100000272National Institute for Health and Care Research (NIHR); and he acknowledges support from the University of Manchester British Heart Foundation Research Excellence Award (RE/24/130017) and the 10.13039/100014653NIHR Manchester Biomedical Research Centre (NIHR203308). Dr Angeli and Dr Laursen were supported by the Danish Research Foundation (“Den danske forskningsfond”); they were partly funded by Nordic Bioscience A/S as PhD candidates at the times of research; immunoassay testing equipment and materials were gifted by Roche Diagnostics International Limited; and Nordic Bioscience A/S performed the sample analysis without charge. Prof Miller has participated on advisory boards/consulted for 10.13039/100004325AstraZeneca, 10.13039/100001003Boehringer Ingelheim and Lilly Alliance, 10.13039/100004336Novartis, and PureTech Health; serves as an advisor for HAYA Therapeutics; has received speaker fees from 10.13039/100004325AstraZeneca, 10.13039/100001003Boehringer Ingelheim, and 10.13039/501100004191Novo Nordisk; has received conference attendance support from 10.13039/100004325AstraZeneca; and research support from 10.13039/100015362Amicus Therapeutics, 10.13039/100004325AstraZeneca, Guerbet Laboratories Limited, Roche, and Univar Solutions B.V. Prof Williams has participated on advisory boards/consulted for 10.13039/100004325AstraZeneca, 10.13039/100004336Novartis, Vifor, and Pharmocosmos; and has received speaker fees from 10.13039/100004325AstraZeneca. Dr Schelbert serves on the Scientific Advisory Board of Haya Therapeutics and sits on the BRITISH Trial Steering Committee. Drs Angeli, Laursen, Karsdal, and Genovese are shareholders and employees at Nordic Bioscience A/S. All other authors have reported that they have no relationships relevant to the contents of this paper to disclose.
