# Supplementary material for: Circulating Endotrophin Predicts Myocardial Fibrosis Burden and Is Sensitive to Antifibrotic Therapy
Source: JACC Adv. 2026 Apr 17;5(5):102738. doi: 10.1016/j.jacadv.2026.102738 (PMC13098592; doi:10.1016/j.jacadv.2026.102738)
Supplement: Supplemental Material [file mmc1.pdf]

Circulating endotrophin predicts myocardial fibrosis burden and is sensitive to anti-fibrotic therapy

Supplemental material

Supplemental Figure 1: Distribution of (A) PRO-C3, (B) C3M, (C) CTX-III, (D) Endotrophin, (E) PRO-C6, and (F) C6M at baseline and 52 weeks.

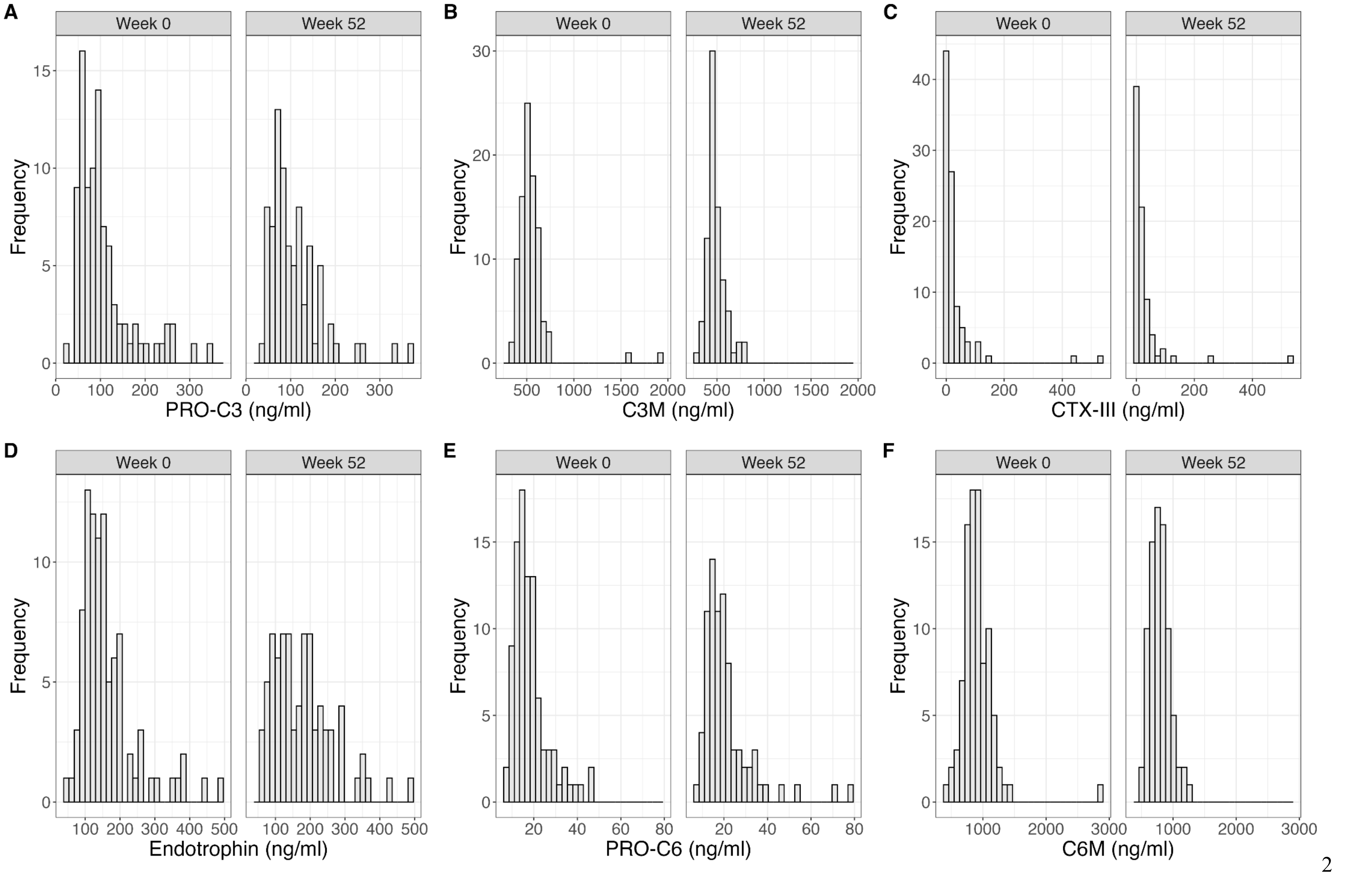

Supplemental Figure 2: Distribution of (A) log PRO-C3, (B) log C3M, (C) log CTX-III, (D) log Endotrophin, (E) log PRO-C6, and (F) log C6M at baseline and 52 weeks.

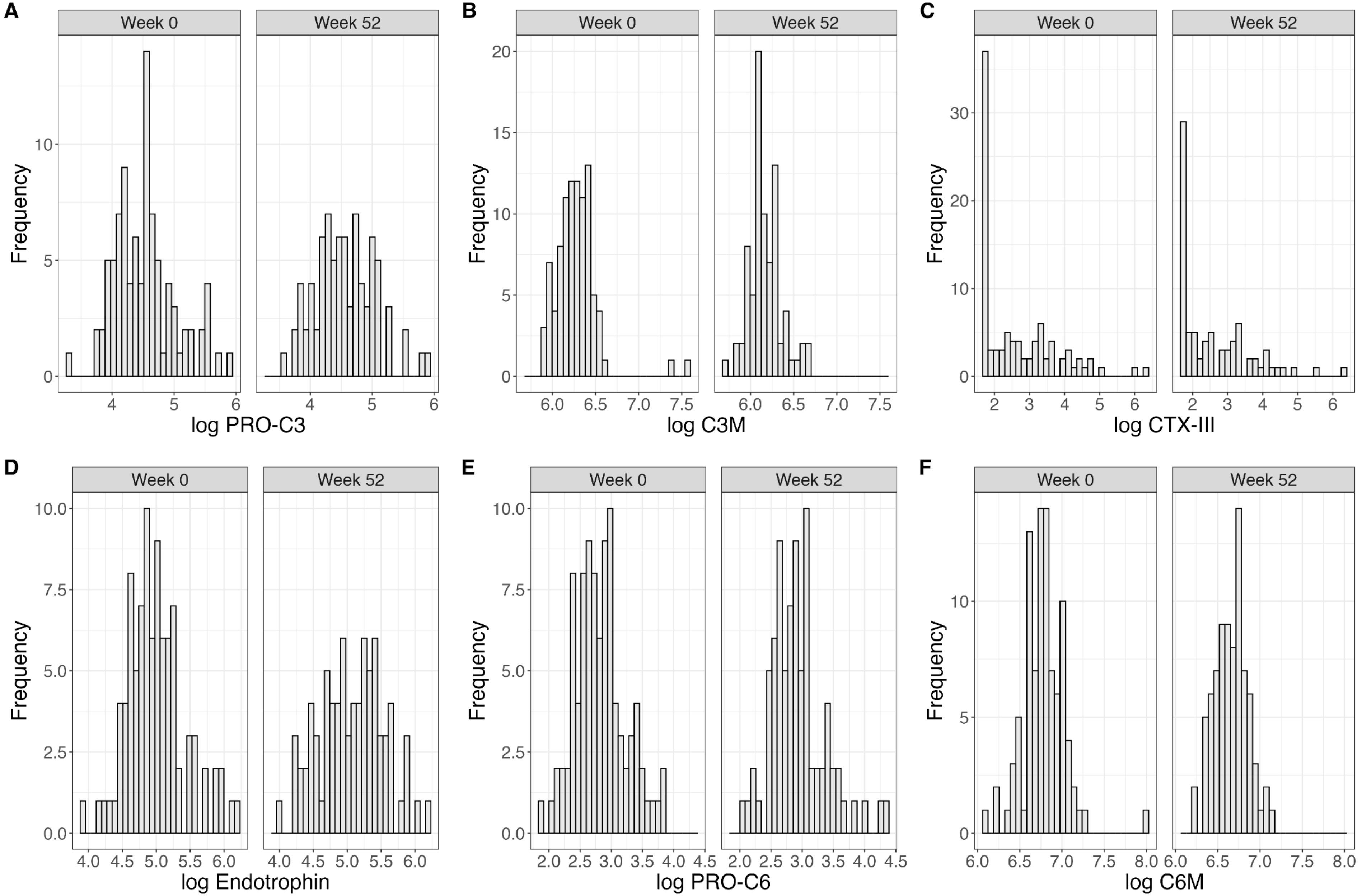

Supplementary Table 1: ANCOVA of week 52 biomarker level. Regression coefficients refer to pirfenidone treatment group.

Biomarker	Regression coefficient (SE)	95% CI	T statistic	P value
log PRO-C3	0.00 (0.10)	-0.20 – 0.19	-0.04	0.970
log C3M	0.01 (0.04)	-0.06 – 0.09	0.37	0.711
log CTX-III	0.07 (0.04)	-0.01 – 0.14	1.82	0.073
log Endotrophin	-0.18 (0.08)	-0.34 - -0.02	-2.16	0.034
log PRO-C6	-0.10 (0.08)	-0.25 – 0.05	-1.26	0.213
log C6M	0.06 (0.04)	-0.03 – 0.15	1.37	0.176

Week 52 ANCOVA adjusted for baseline biomarker level, sex and pirfenidone treatment group. Regression coefficients refer to pirfenidone treatment group.

ANCOVA = analysis of covariance, CI = confidence interval, SE = standard error

Supplementary Table 2: ANCOVA of week 52 biomarker level with additional adjustment for BMI and eGFR. Regression coefficients refer to pirfenidone treatment group.

Biomarker	Regression coefficient (SE)	95% CI	T statistic	P value
log PRO-C3	-0.03 (0.10)	-0.23 – 0.18	-0.26	0.797
log C3M	0.02 (0.04)	-0.06 – 0.09	0.42	0.675
log CTX-III	0.07 (0.04)	-0.01 – 0.00	1.79	0.078
log Endotrophin	-0.17 (0.08)	-0.34 - -0.01	-2.12	0.037
log PRO-C6	-0.08 (0.07)	-0.23 – 0.07	-1.08	0.282
log C6M	0.06 (0.04)	-0.01 – 0.01	1.24	0.221

Week 52 ANCOVA adjusted for baseline biomarker level, sex, pirfenidone treatment group, BMI and eGFR. Regression coefficients refer to pirfenidone treatment group.

ANCOVA = analysis of covariance, CI = confidence interval, SE = standard error

Supplementary Table 3: Repeated measures linear mixed model. Regression coefficients refer to pirfenidone treatment group and interaction between time and treatment group.

Biomarker	Pirfenidone treatment group				Interaction between pirfenidone treatment group and time			
	**Regression coefficient (SE)**	**95% CI**	**T statistic**	**P value**	**Regression coefficient (SE)**	**95% CI**	**T statistic**	**P value**
log PRO-C3	-0.04 (0.13)	-0.28 – 0.19	-0.37	0.713	0.00 (0.01)	-0.02 – 0.03	0.19	0.846
log C3M	-0.08 (0.05)	-0.18 – 0.02	-1.63	0.107	0.01 (0.01)	0.00 – 0.02	1.61	0.109
log CTX-III	-0.30 (0.29)	-0.88 – 0.28	-1.02	0.311	0.02 (0.03)	-0.03 – 0.08	0.76	0.447
log Endotrophin	-0.24 (0.09)	-0.43 - -0.05	-2.53	0.014	0.00 (0.01)	-0.02 – 0.03	0.18	0.858
log PRO-C6	-0.07 (0.07)	-0.22 – 0.07	-1.01	0.316	0.00 (0.01)	-0.02 – 0.02	-0.10	0.922
log C6M	-0.03 (0.06)	-0.15 – 0.09	-0.50	0.619	0.01 (0.01)	-0.01 – 0.02	1.20	0.233

Repeated measures linear mixed model adjusted for time (months), baseline biomarker levels, sex, pirfenidone treatment group and interaction between time and pirfenidone treatment group.

CI = confidence interval, SE = standard error

Supplementary Table 4: Univariable associations between change in myocardial ECV from baseline to week 52, change in log biomarkers, and baseline characteristics.

Variable	Univariable associations		
	Regression coefficient (SE)	T statistic	P value
Age (year)*	0.11 (0.25)	0.46	0.647
Aorta distensibility (10^{-3}/mmHg)*	0.04 (0.24)	0.17	0.864
AF	0.86 (0.46)	1.87	0.065
BMI (kg/m^2)*	0.13 (0.24)	0.52	0.601
**Change in log PRO-C3***	**-0.11 (0.24)**	**-0.47**	**0.637**
**Change in log C3M***	**-0.08 (0.24)**	**-0.35**	**0.731**
**Change in log CTX-III***	**-0.34 (0.23)**	**-1.46**	**0.150**
**Change in log Endotrophin***	**0.40 (0.23)**	**1.74**	**0.069**
**Change in log PRO-C6***	**0.43 (0.23)**	**1.85**	**0.087**
**Change in log C6M***	**-0.31 (0.23)**	**-1.35**	**0.182**
Change in HsTnT (pg/ml)*	-0.18 (0.24)	-0.74	0.459
Change in log NT-proBNP*	0.24 (0.23)	1.02	0.312
Change in log GDF-15*	0.36 (0.23)	1.57	0.121
COPD	0.08 (0.70)	0.11	0.911
Diabetes	0.67 (0.53)	1.25	0.214
Ex-smoker	0.77 (0.49)	1.58	0.117
Female sex	-0.71 (0.46)	-1.55	0.126
GFR (ml/min/1.73m^2)*	-0.02 (0.23)	-0.10	0.918
Haemoglobin (g/dL)*	0.03 (0.24)	0.13	0.895
Hyperlipidaemia	0.29 (0.54)	0.53	0.596
Hypertension	0.13 (0.63)	0.20	0.841
Infarct LGE	0.30 (0.59)	0.51	0.612
Ischaemic heart disease	-0.03 (0.48)	-0.07	0.945
LAVi (ml/m^2)*	0.31 (0.22)	1.41	0.164
LVEDVi (ml/m^2)*	-0.14 (0.22)	-0.64	0.527
LVEF (%)*	-0.35 (0.23)	-1.51	0.134
LVMassi (g)*	0.47 (0.22)	2.10	0.039
Non-white ethnicity	-1.00 (0.95)	-1.06	0.295
Prior HF hospitalisation	0.81 (0.77)	1.05	0.295
RAVI (ml/m^2)*	0.05 (0.22)	0.21	0.835
RVEDVi (ml/m^2)*	-0.06 (0.24)	-0.23	0.817
RVEF (%)*	-0.30 (0.23)	-1.29	0.200
Sodium (mmol/L)*	0.10 (0.24)	0.42	0.676
Stroke	0.40 (0.73)	0.55	0.585
White cell count (10^9/L)*	0.36 (0.23)	1.56	0.122

*Regression coefficients standardised to 1 standard deviation change in continuous variables

AF = atrial fibrillation, BMI = body mass index, COPD = chronic obstructive pulmonary disease, ECV = extracellular volume, GDF-15 = growth differentiation factor 15, GFR = glomerular filtration rate, HsTnT = high sensitivity troponin T, LGE = late gadolinium enhancement, LAVi = indexed left atrial volume, LVEDVi = indexed left ventricle end diastolic volume, LVEF = left ventricle ejection fraction, LVMassi = indexed left ventricle mass, NT-proBNP = N-terminal pro B-type natriuretic peptide, HF = heart failure, RAVi = indexed right atrial volume, RVEDVi = indexed right ventricle end diastolic volume, RVEF = right ventricle ejection fraction, SE=standard error

Supplementary Table 5: Multivariable adjusted associations between change in myocardial ECV and change in log Endotrophin from baseline to week 52. Multivariable associations without inclusion of Endotrophin included for comparison.

Variable	Multivariable associations (Adjusted R^2 0.14)			Multivariable associations without Endotrophin (Adjusted R^2 0.10)		
	**Regression coefficient (SE)**	**T statistic**	**P value**	**Regression coefficient (SE)**	**T statistic**	**P value**
Intercept	-0.88 (0.36)	-2.46	0.017	-0.78 (0.36)	-2.15	0.035
**Change in log Endotrophin***	**0.50 (0.23)**	**2.21**	**0.031**	–	–	–
Atrial fibrillation	0.90 (0.45)	2.02	0.047	0.86 (0.45)	1.91	0.060
Ex-smoker	0.88 (0.48)	1.82	0.072	0.64 (0.48)	1.35	0.182
Change in log GDF-15*	0.35 (0.22)	1.58	0.118	0.37 (0.22)	1.66	0.100
Left ventricular mass indexed (g/m^2)*	0.39 (0.22)	1.75	0.085	0.43 (0.22)	1.91	0.060

*Regression coefficients standardised to 1 standard deviation change in continuous variables

ECV=extracellular volume, SE=standard error

Supplementary Table 6: Multivariable adjusted associations between change in myocardial ECV and change in log PRO-C3 from baseline to week 52.

**Variable**	**Multivariable associations (Adjusted R^2 0.10)**		
	**Regression coefficient (SE)**	**T statistic**	**P value**
Intercept	-0.54 (0.34)	-1.59	0.117
**Change in log PRO-C3***	**-0.21 (0.23)**	**-0.92**	**0.362**
Atrial fibrillation	0.87 (0.46)	1.91	0.061
Change in log GDF-15*	0.37 (0.22)	1.63	0.108
Left ventricular mass indexed (g/m^2)*	0.47 (0.23)	2.09	0.041
White cell count ($x10^9/L$)*	0.34 (0.23)	1.49	0.140

*Regression coefficients standardised to 1 standard deviation change in continuous variables

ECV=extracellular volume, SE=standard error

Supplementary Table 7: Multivariable adjusted associations between change in myocardial ECV and change in log C3M from baseline to week 52.

**Variable**	**Multivariable associations (Adjusted R^2 0.09)**		
	**Regression coefficient (SE)**	**T statistic**	**P value**
Intercept	-0.57 (0.34)	-1.67	0.099
**Change in log C3M***	**-0.13 (0.24)**	**-0.56**	**0.577**
Atrial fibrillation	0.89 (0.45)	1.97	0.052
Change in log GDF-15*	0.39 (0.23)	1.70	0.093
Left ventricular mass indexed (g/m^2)*	0.44 (0.22)	1.98	0.052
White cell count ($x10^9/L$)*	0.29 (0.23)	1.28	0.205

*Regression coefficients standardised to 1 standard deviation change in continuous variables

ECV=extracellular volume, SE=standard error

Supplementary Table 8: Multivariable adjusted associations between change in myocardial ECV and change in log CTX-III from baseline to week 52.

Variable	Multivariable associations (Adjusted R^2 0.11)		
	**Regression coefficient (SE)**	**T statistic**	**P value**
Intercept	-0.61 (0.34)	-1.81	0.075
**Change in log CTX-III***	**-0.34 (0.22)**	**-1.55**	**0.126**
Atrial fibrillation	0.94 (0.45)	2.11	0.039
Change in log GDF-15*	0.37 (0.22)	1.66	0.100
Left ventricular mass indexed (g/m^2)*	0.44 (0.22)	1.97	0.053

*Regression coefficients standardised to 1 standard deviation change in continuous variables

ECV=extracellular volume, SE=standard error

Supplementary Table 9: Multivariable adjusted associations between change in myocardial ECV and change in log PRO-C6 from baseline to week 52.

Variable	Multivariable associations (Adjusted R^2 0.11)		
	**Regression coefficient (SE)**	**T statistic**	**P value**
Intercept	-0.68 (0.36)	-1.86	0.067
**Change in log PRO-C6***	**0.38 (0.22)**	**1.72**	**0.090**
Atrial fibrillation	0.83 (0.45)	1.85	0.068
Diabetes	0.85 (0.52)	1.65	0.103
Left ventricular mass indexed (g/m^2)*	0.47 (0.22)	2.12	0.037
Non-white ethnicity	-1.30 (0.93)	-1.41	0.163

*Regression coefficients standardised to 1 standard deviation change in continuous variables

ECV=extracellular volume, SE=standard error

Supplementary Table 10: Multivariable adjusted associations between change in myocardial ECV and change in log C6M from baseline to week 52.

**Variable**	**Multivariable associations (Adjusted R^2 0.10)**		
	**Regression coefficient (SE)**	**T statistic**	**P value**
Intercept	-0.59 (0.34)	-1.73	0.087
**Change in log C6M***	**-0.21 (0.23)**	**-0.90**	**0.373**
Atrial fibrillation	0.93 (0.45)	2.05	0.045
Change in log GDF-15*	0.34 (0.22)	1.54	0.129
Left ventricular mass indexed (g/m^2)*	0.42 (0.22)	1.88	0.064
White cell count ($x10^9/L$)*	0.26 (0.23)	1.13	0.261

*Regression coefficients standardised to 1 standard deviation change in continuous variables

ECV=extracellular volume, SE=standard error

Supplementary Table 11: Univariable associations between baseline myocardial ECV and baseline characteristics.

**Variable**	**Univariable associations**		
	**Regression coefficient (SE)**	**T statistic**	**P value**
Age (year)*	0.00 (0.29)	-0.01	0.994
Aorta distensibility (10^{-3}/mmHg)*	0.03 (0.29)	0.09	0.925
AF	0.95 (0.57)	1.68	0.097
BMI (kg/m^2)*	-0.76 (0.27)	-2.78	0.007
**log PRO-C3***	**0.02 (0.29)**	**0.08**	**0.935**
**log C3M***	**-0.20 (0.29)**	**-0.70**	**0.486**
**log CTX-III***	**0.36 (0.29)**	**1.24**	**0.217**
**log Endotrophin***	**0.60 (0.28)**	**2.15**	**0.034**
**log PRO-C6***	**0.63 (0.28)**	**2.23**	**0.028**
**log C6M***	**-0.11 (0.29)**	**-0.38**	**0.708**
COPD	0.45 (0.85)	0.53	0.600
Current smoker	-1.20 (2.77)	-0.43	0.667
Diabetes	-0.40 (0.62)	-0.65	0.516
Ex-smoker	-0.08 (0.60)	-0.13	0.894
Female sex	-0.66 (0.57)	-1.17	0.244
log GDF-15*	0.48 (0.28)	1.72	0.089
GFR (ml/min/1.73m^2)*	-0.06 (0.29)	-0.21	0.837
Haemoglobin (g/dL)*	-0.56 (0.28)	-1.98	0.050
Hyperlipidaemia	-0.94 (0.66)	-1.41	0.160
Hypertension	0.12 (0.78)	0.16	0.874
HsTnT (pg/ml)*	0.66 (0.28)	2.39	0.019
Infarct LGE	-0.02 (0.70)	-0.03	0.972
Ischaemic heart disease	1.04 (0.58)	1.81	0.074
LAVi (ml/m^2)*	0.70 (0.28)	2.53	0.013
LVEDVi (ml/m^2)*	0.01 (0.29)	0.04	0.965
LVEF (%)*	0.17 (0.29)	0.60	0.549
LVMassi (g)*	0.52 (0.28)	1.84	0.068
Non-white ethnicity	0.57 (1.16)	0.49	0.626
log NT-proBNP*	0.98 (0.27)	3.69	<0.001
Prior HF hospitalisation	-0.20 (0.78)	-0.25	0.802
RAVI (ml/m^2)*	0.62 (0.28)	2.23	0.028
RVEDVi (ml/m^2)*	0.24 (0.28)	0.86	0.394
RVEF (%)*	0.02 (0.29)	0.08	0.934
Sodium (mmol/L)*	-0.27 (0.28)	-0.96	0.340
Stroke	1.75 (0.90)	1.94	0.056
White cell count (10^9/L)*	-0.36 (0.28)	-1.28	0.202

*Regression coefficients standardised to 1 standard deviation change in continuous variables

AF = atrial fibrillation, BMI = body mass index, COPD = chronic obstructive pulmonary disease, ECV = extracellular volume, GDF-15 = growth differentiation factor 15, GFR = glomerular filtration rate, HsTnT = high sensitivity troponin T, LGE = late gadolinium enhancement, LAVi = indexed left atrial volume, LVEDVi = indexed left ventricle end diastolic volume, LVEF = left ventricle ejection fraction, LVMassi = indexed left ventricle mass, NT-proBNP = N-terminal pro B-type natriuretic peptide, HF = heart failure, RAVi = indexed right atrial volume, RVEDVi = indexed right ventricle end diastolic volume, RVEF = right ventricle ejection fraction, SE=standard error

Supplementary Table 12: Multivariable adjusted associations between baseline myocardial ECV and baseline log Endotrophin. Multivariable associations without inclusion of Endotrophin included for comparison.

Variable	Multivariable associations (Adjusted R^2 0.30)			Multivariable associations without Endotrophin (Adjusted R^2 0.26)		
	**Regression coefficient (SE)**	**T statistic**	**P value**	**Regression coefficient (SE)**	**T statistic**	**P value**
Intercept	29.18 (0.40)	72.40	<0.001	29.17 (0.41)	70.57	<0.001
**log Endotrophin***	**0.60 (0.26)**	**2.33**	**0.022**	–	–	–
AF	1.72 (0.51)	3.39	0.001	1.76 (0.52)	3.39	0.001
BMI (kg/m^2)*	-0.78 (0.25)	-3.10	0.003	-0.78 (0.26)	-3.01	0.003
Haemoglobin (g/dL)*	-0.66 (0.26)	-2.51	0.014	-0.86 (0.25)	-3.39	0.001
Hyperlipidaemia	-1.30 (0.58)	-2.22	0.029	-1.29 (0.60)	-2.15	0.034
LVmassi (g/m^2)*	0.65 (0.25)	2.64	0.010	0.57 (0.25)	2.28	0.025
Stroke	2.17 (0.82)	2.65	0.010	2.01 (0.84)	2.41	0.018

*Regression coefficients standardised to 1 standard deviation change in continuous variables

AF=atrial fibrillation, BMI=body mass index, ECV=extracellular volume, LVmassi=indexed left ventricular mass, SE=standard error

Supplementary Table 13: Multivariable adjusted associations between baseline myocardial ECV and baseline log PRO-C3.

Variable	Multivariable associations (Adjusted R^2 0.27)		
	**Regression coefficient (SE)**	**T statistic**	**P value**
Intercept	29.53 (0.45)	65.78	<0.001
**log PRO-C3***	**-0.06 (0.26)**	**-0.24**	**0.813**
AF	1.12 (0.62)	1.80	0.075
BMI (kg/m^2)*	-0.65 (0.27)	-2.43	0.017
Haemoglobin (g/dL)*	-0.77 (0.26)	-2.98	0.004
Hyperlipidaemia	-1.20 (0.60)	-1.99	0.050
LVmassi (g/m^2)*	0.50 (0.25)	2.01	0.048
log NT-proBNP*	0.60 (0.30)	1.99	0.050
Stroke	1.92 (0.85)	2.27	0.026

*Regression coefficients standardised to 1 standard deviation change in continuous variables

AF=atrial fibrillation, BMI=body mass index, ECV=extracellular volume, LVmassi=indexed left ventricular mass, SE=standard error

Supplementary Table 14: Multivariable adjusted associations between baseline myocardial ECV and baseline log C3M.

**Variable**	**Multivariable associations (Adjusted R^2 0.28)**		
	**Regression coefficient (SE)**	**T statistic**	**P value**
Intercept	29.49 (0.44)	66.38	<0.001
**log C3M***	**0.23 (0.26)**	**0.87**	**0.385**
AF	1.12 (0.61)	1.83	0.072
BMI (kg/m^2)*	-0.72 (0.27)	-2.65	0.010
Haemoglobin (g/dL)*	-0.79 (0.25)	-3.12	0.002
Hyperlipidaemia	-1.18 (0.59)	-1.98	0.051
LVmassi (g/m^2)*	0.53 (0.25)	2.12	0.037
log NT-proBNP*	0.60 (0.30)	2.01	0.048
Stroke	2.10 (0.84)	2.51	0.014

*Regression coefficients standardised to 1 standard deviation change in continuous variables

AF=atrial fibrillation, BMI=body mass index, ECV=extracellular volume, LVmassi=indexed left ventricular mass, NT-proBNP=N-terminal pro B-type natriuretic peptide, SE=standard error

Supplementary Table 15: Multivariable adjusted associations between baseline myocardial ECV and baseline log CTX-III.

Variable	Multivariable associations (Adjusted R^2 0.28)		
	**Regression coefficient (SE)**	**T statistic**	**P value**
Intercept	29.53 (0.45)	65.88	<0.001
**log CTX-III***	**0.08 (0.27)**	**0.29**	**0.770**
AF	1.10 (0.62)	1.78	0.079
BMI (kg/m^2)*	-0.66 (0.26)	-2.49	0.015
Haemoglobin (g/dL)*	-0.78 (0.26)	-2.96	0.004
Hyperlipidaemia	-1.20 (0.61)	-1.96	0.053
LVmassi (g/m^2)*	0.51 (0.25)	2.06	0.042
log NT-proBNP*	0.56 (0.30)	1.86	0.067
Stroke	1.99 (0.83)	2.40	0.019

*Regression coefficients standardised to 1 standard deviation change in continuous variables

AF=atrial fibrillation, BMI=body mass index, ECV=extracellular volume, LVmassi=indexed left ventricular mass, NT-proBNP=N-terminal pro B-type natriuretic peptide, SE=standard error

Supplementary Table 16: Multivariable adjusted associations between baseline myocardial ECV and baseline log PRO-C6.

Variable	Multivariable associations (Adjusted R^2 0.29)		
	**Regression coefficient (SE)**	**T statistic**	**P value**
Intercept	29.53 (0.44)	66.59	<0.001
**log PRO-C6***	**0.30 (0.29)**	**1.03**	**0.306**
AF	1.07 (0.61)	1.76	0.083
BMI (kg/m^2)*	-0.67 (0.26)	0.256	0.012
Haemoglobin (g/dL)*	-0.69 (0.28)	-2.50	0.015
Hyperlipidaemia	-1.20 (0.59)	-2.02	0.047
LVmassi (g/m^2)*	0.54 (0.25)	2.17	0.033
log NT-proBNP*	0.47 (0.31)	1.50	0.137
Stroke	2.08 (0.83)	2.51	0.014

*Regression coefficients standardised to 1 standard deviation change in continuous variables

AF=atrial fibrillation, BMI=body mass index, ECV=extracellular volume, LVmassi=indexed left ventricular mass, NT-proBNP=N-terminal pro B-type natriuretic peptide, SE=standard error

Supplementary Table 17: Multivariable adjusted associations between baseline myocardial ECV and baseline log C6M.

Variable	Multivariable associations (Adjusted R^2 0.28)		
	**Regression coefficient (SE)**	**T statistic**	**P value**
Intercept	29.48 (0.45)	65.76	<0.001
**log C6M***	**0.16 (0.27)**	**0.60**	**0.554**
AF	1.18 (0.63)	1.88	0.064
BMI (kg/m^2)*	-0.67 (0.26)	-2.54	0.013
Haemoglobin (g/dL)*	-0.82 (0.25)	-3.22	0.002
Hyperlipidaemia	-1.26 (0.62)	-2.04	0.045
LVmassi (g/m^2)*	0.49 (0.25)	1.93	0.057
log NT-proBNP*	0.56 (0.30)	1.85	0.068
Stroke	2.12 (0.86)	2.46	0.016

*Regression coefficients standardised to 1 standard deviation change in continuous variables

AF=atrial fibrillation, BMI=body mass index, ECV=extracellular volume, LVmassi=indexed left ventricular mass, NT-proBNP=N-terminal pro B-type natriuretic peptide, SE=standard error

Supplementary Table 18: Levels of circulating biomarkers in randomised vs registry patients. P value for two-sample t test.

	**Randomised (n=94)**	**Registry (n=13)**	**T statistic**	**P value**
log PRO-C3	4.53 (0.49)	4.11 (0.28)	-2.99	0.004
log C3M	6.27 (0.24)	6.29 (0.25)	-0.23	0.815
log CTX-III	2.63 (1.08)	2.55 (1.21)	0.25	0.801
log Endotrophin	4.98 (0.43)	4.92 (0.41)	0.54	0.590
log PRO-C6	2.83 (0.40)	2.67 (0.26)	1.41	0.163
log C6M	6.78 (0.25)	6.74 (0.21)	0.55	0.585

Values are mean ± (standard deviation)
